# Experimental Study on Bond Performance Between Negative Poisson’s Ratio of Bars/Steel Strands and Concrete

**DOI:** 10.3390/ma19112221

**Published:** 2026-05-25

**Authors:** Qing Wu, Feiyan Zhang, Zonghua Wu, Yunzhou Chen, Huiqiang Zhao, Xiang Liu

**Affiliations:** 1 School of Management Engineering, Zhejiang Guansha Vocational and Technical University of Construction, Dongyang 322100, China; 20191929@zjgsdx.edu.cn (Q.W.); 20191932@zjgsdx.edu.cn (Z.W.); chenyunzhou1990@zjgsdx.edu.cn (Y.C.); 2 College of Civil Engineering, Fujian University of Technology, Fuzhou 350118, China; zhaohuiqiang0905@163.com

**Keywords:** NPR bar, bond performance, failure mode, bond–slip constitutive model

## Abstract

Negative Poisson’s ratio (NPR) bars, as novel materials, exhibit a significant volumetric dilation effect under tension. Compared to conventional reinforcement, NPR bars offer distinct advantages, including high ductility, high strength, and superior corrosion resistance. This study investigates the tensile properties of three types of NPR bars: the bare round bar, spiral ribbed bar, and steel strand. Their bond behavior with concrete was examined through central pull-out tests, considering the influences of bar type, NPR bar diameter, and anchorage length. The analysis focuses on the tensile mechanical properties, characteristics of the bond–slip curves, failure modes, and the development of predictive models for key bond–slip parameters. The results indicate that all three NPR types possess a high elastic modulus and exceptional ductility. The bare round bar achieved an elongation at break of 51.2%, with only minor necking observed at the fracture surface. The bond failure mode is influenced by bar type, NPR bar diameter, and anchorage length: pull-out failure occurred for the bare round bar, spiral ribbed bar with short anchorage length, and small-diameter steel strand, whereas splitting failure was observed for the spiral ribbed bar with long anchorage length. The large-diameter strand exhibited a combined splitting–pull-out failure. Furthermore, the bond–slip curves for the bare round bar and steel strand displayed two distinct peak strengths. The bond strength of the bare round bar increased with longer anchorage length, while it decreased for both the spiral ribbed bar and steel strand. Empirical models developed based on experimental data demonstrate good predictive accuracy for the bond performance of the different bar types.

## 1. Introduction

Reinforced concrete (RC) structures are widely employed in various civil engineering applications, including buildings, bridges, and roads, due to the continuous development of the construction industry [1]. The strength of RC lies in the complementary combination of concrete’s superior compressive strength and steel reinforcement’s excellent tensile capacity [2]. However, conventional steel reinforcement is susceptible to early necking under large tensile deformation, which severely reduces its ductility—a crucial drawback under extreme loading conditions such as earthquakes [3]. To address this deficiency, He and his team developed novel negative Poisson’s ratio (NPR) steel reinforcement [4].

**Figure 1:**
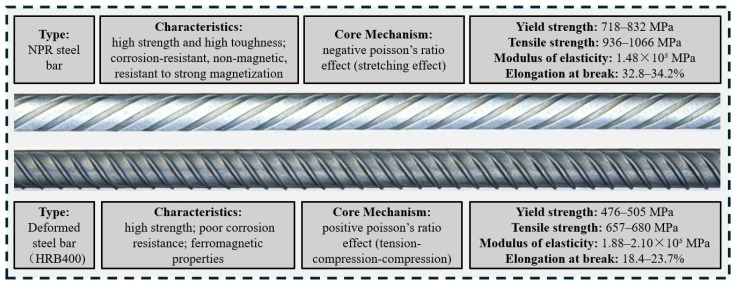
Comparison of the NPR steel bar and HRB400 steel bar.

Figure 1 compares the differences between NPR bars and HRB400 grade steel bars. NPR bars exhibit superior ductility, enhanced corrosion resistance, and reduced susceptibility to magnetization compared to traditional steel bars [5]. During the stretching process, NPR steel demonstrates a remarkable and sustained ability to harden under strain due to the combined effects of continuous mechanical twinning and the transformation-induced plasticity of retained austenite, resulting in a superior uniform elongation rate [6]. In the work of Wang et al. [7], the dynamic models of NPR bolts were analyzed, and a validated three-stage dynamic model (elastic, slipping, and recovery) showed that NPR bolts reduce peak impact force by 10.6% and avoid brittle fracture (model error < 7%). The plastic deformation in NPR bars is distributed extensively along the axis, resulting in a gradual change in the fracture profile and more uniform elongation. Unlike conventional steel, which shows negligible volume change during plastic deformation, NPR bars exhibit volumetric expansion in this phase [6]. Currently, due to higher manufacturing costs, NPR bars are primarily limited to specialized fields such as aerospace and medicine [8].

The bond performance between steel reinforcement and concrete is the foundation for their collaborative work. When unconventional reinforcements, such as FRP bars, are used, their bond behavior with concrete has always been a major concern [9]. Given the negative Poisson’s ratio characteristic, NPR bars undergo lateral expansion upon axial tension, suggesting that their bond–slip failure mechanism may fundamentally differ from that of conventional steel–concrete systems. Consequently, numerous studies have investigated the bond behavior between NPR bars and concrete, with a summary of recent findings presented in Table 1. Notably, Xu et al. [5] studied the bonding characteristics of NPR bars with Ultra-High-Performance Concrete (UHPC), concluding that existing models underestimate the bond strength. They proposed an Artificial Neural Network (ANN) model with an R^2^ exceeding 0.90 for accurate prediction of key bond parameters.

The work by Lu et al. [10] further demonstrated that NPR bars embedded in UHPC achieved a 21% higher ultimate bond strength than HRB400 bars but a 13% lower bond strength than HRB635 bars, identifying three distinct failure modes. Using eccentric pull-out tests, Xu et al. [14] found that although the bond performance of NPR bars in UHPC was superior to that of HRB400 bars, it decreased with increasing pre-strain; a 22.0% pre-strain reduced the bond strength by 17.01%. Shao et al. [11] investigated the bond performance of NPR bars in marine concrete, reporting that their bond strength was approximately 25% lower than that of HRB400 bars. However, increasing the concrete strength from C30 to C50 resulted in an up to 40% enhancement in bond strength. Xiong et al. [12] found that NPR bars with six helical grooves exhibited higher bond strength than those with three grooves, and the strength decreased as the anchor length increased. While reducing stirrup spacing improved bond strength, the effect was limited. Stirrups also helped to moderate the descending branch of the bond–slip curve, improve the failure mode, and enhance ductility and energy absorption. Zhou et al. [13] investigated the effect of corrosion, observing that the bond strength of corroded NPR bars in marine concrete first increased and then decreased with the corrosion rate, peaking at an actual corrosion rate of approximately 1.8%. Focusing on bond performance under stirrup confinement, Li et al. [15] determined that increasing the stirrup ratio enhances the ultimate pull-out force and established 41 times the bar diameter as the critical anchorage length. Regarding the mechanical performance and durability of members, Lu et al. [3] investigated bond behavior under cyclic loading, confirming that NPR bars exhibit superior energy dissipation capacity and fatigue resistance compared to HRB635, HRB400, and HG reinforcement.

Furthermore, the performance of structural members reinforced with NPR bars has garnered considerable attention. Specifically, the study by Long et al. [16] on NPR-UHPC beams indicated that these beams maintained a drift ratio exceeding 8% under cyclic loading, demonstrating extremely high-load-bearing and energy dissipation capacities. Guo et al. [17] also investigated the flexural behavior of NPR-UHPC beams, showing that the inclusion of NPR bars significantly enhanced the flexural performance of the UHPC beams. The flexural strength was improved by 47.8–55.2%, and the deformation capacity and toughness increased by 13.6–180.9%. Gu et al. [18] tested the NPR steel rebar (1100 MPa, 40% elongation) in UHPC beams under cyclic loading. Compared to HRB400, the NPR rebar increased flexural capacity by 47.8% (monotonic) and 52.3% (cyclic), improved energy dissipation and stiffness control, and produced 7–8 fine cracks in the pure bending zone.

In geotechnical engineering applications, indoor shear tests by He et al. [4] confirmed that quasi-NPR bar anchors surpass conventional anchors in shear deformation capacity, strength contribution, and energy absorption characteristics by 3.5 times, nearly 2 times, and over 3.68 times, respectively, demonstrating immense application potential.

In summary, previous studies on the bond behavior of NPR bars have predominantly focused on a single bar type (e.g., spiral-grooved bars) in specific concrete matrices (e.g., UHPC or marine concrete), with limited attention given to the systematic comparison of different surface configurations, diameters, and anchorage lengths. Moreover, a predictive bond model that captures the coupled effects of concrete strength, bar diameter, and bond length for NPR bars remains absent. Therefore, this paper discussed the bond behavior of three types of normal concrete NPR bars, including bare round bars, spiral ribbed bars, and steel strands.

## 2. Experimental Programs

### 2.1. Raw Materials

#### 2.1.1. NPR Bars

**Figure 2:**
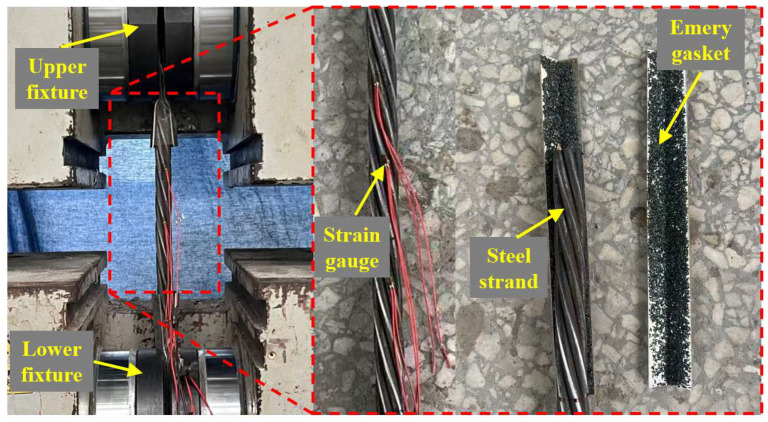
Bar tensile experimental setup.

The NPR bars used in this test comprised three distinct types: the bare round bar, the spiral ribbed bar, and the steel strand. The bare round bar had a bar diameter of 9.6 mm; the spiral ribbed bar was 18 mm in bar diameter; and the steel strands were tested in two bar diameters: 15.2 mm and 21.6 mm. Tensile tests were conducted on the four different types of NPR bars at room temperature using a 100T universal testing machine (MTS Industrial Systems Co., Ltd., Eden Prairie, USA), in accordance with the standard GB/T 228.1-2010 [19]. Strain gauges were affixed to the middle section of the bars to ensure accurate strain measurement. Abrasive pads were utilized to secure the NPR steel strands and prevent slippage during testing, as shown in Figure 2.

**Figure 3:**
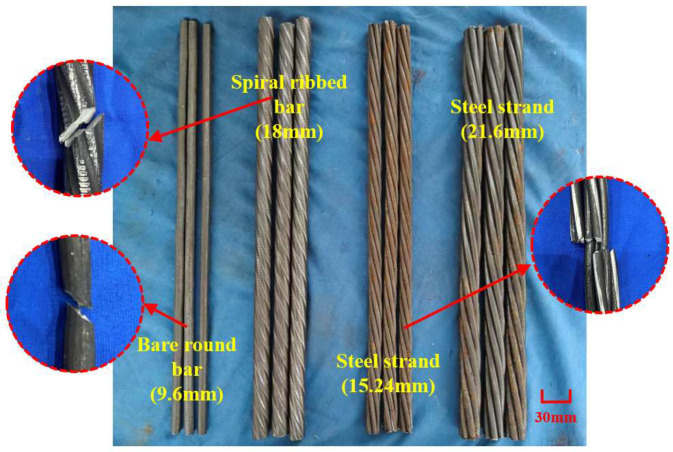
Physical and damage characteristics of NPR bars with different surface properties.

The test results are summarized in Table 2, with the physical samples and failure modes presented in Figure 3. The NPR bar’s negative Poisson’s ratio effect proved beneficial during tensile fracture by effectively suppressing the development of necking. The spiral ribbed bar and steel strand showed minimal necking and maintained a relatively intact cross-section, while the smooth NPR bar, due to its smaller diameter, exhibited a relatively more noticeable diameter reduction.

**Figure 4:**
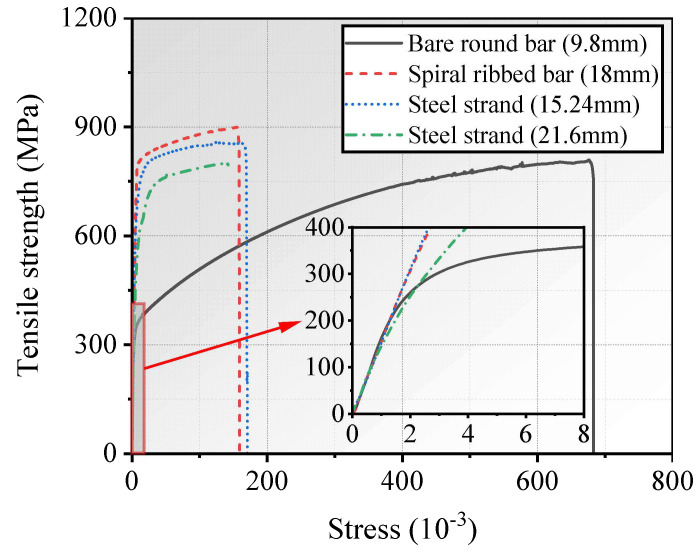
Stress–strain curve of NPR bars.

The stress–strain curves for the four types of bars are displayed in Figure 4. The stress–strain relationship of the new NPR bars primarily consists of two parts: an elastic stage and a hardening stage. No distinct yield point was observed. The spiral ribbed bar and steel strand showed similar stress–strain behavior, fracturing before the strain reached 0.2, with an ultimate strength of approximately 850 MPa and an elongation of about 20%. In contrast, the bare round bar demonstrated much greater ductility, with a fracture strain of about 0.7, resulting in an elongation of 51.2% and a fracture stress of 806 MPa. All three NPR bars exhibited similar tensile stiffness, with an elastic modulus of approximately 150 GPa.

#### 2.1.2. Concrete

**Figure 5:**
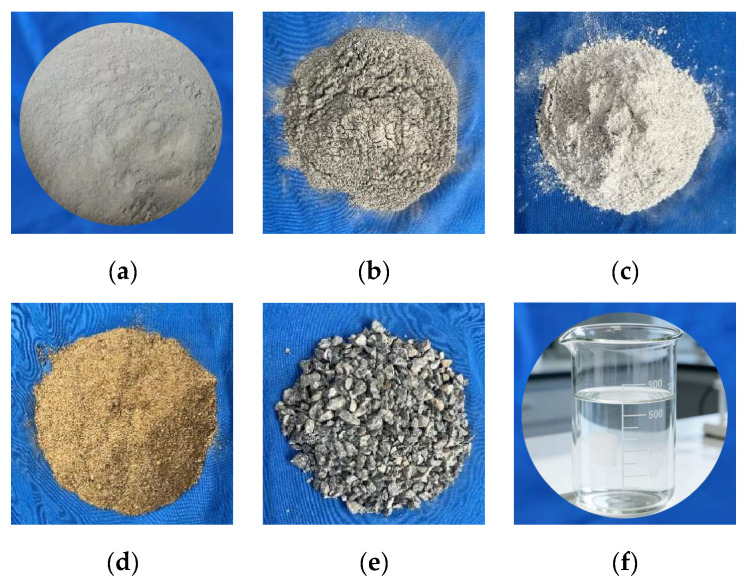
Concrete raw materials: (**a**) GBFS; (**b**) cement; (**c**) FA; (**d**) sand; (**e**) stone; (**f**) water.

The concrete specimens were prepared using P.O. 42.5 Portland cement, S95-grade Ground Granulated Blast-Furnace Slag (GBFS), and Class F Grade I fly ash (FA). River sand (fineness modulus 2.8) and gravel (particle size 5–20 mm) were sourced from a quarry in Fujian. To enhance the mixture’s workability, a polycarboxylate superplasticizer with a water reduction rate of 45% was added during mixing. The physical components are shown in Figure 5.

### 2.2. Mix Proportions and Specimen Preparation

#### 2.2.1. Mixing Ratio Design

**Figure 6:**
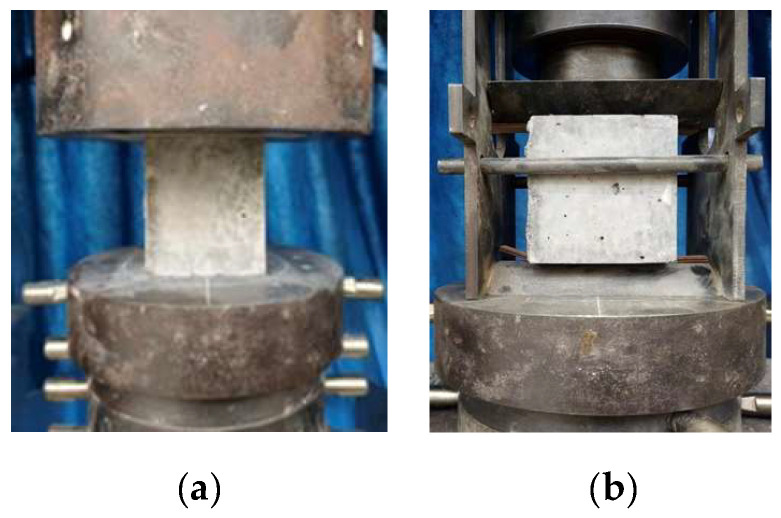
Mechanical performance test: (**a**) compressive test; (**b**) splitting tensile test.

Normal concrete with a target strength of C50 was designed, and its mix proportion is presented in Table 3. During the preparation of the concrete, the slump was measured. Twelve 150 mm cubes were cast to determine the compressive strength and tensile strength was split at the ages of 7 and 28 days, and three blocks were tested for each group. According to 20. GB/T 50080-2016 [20], loading tests were conducted using a universal testing machine at a rate of 0.5 MPa/s, as shown in Figure 6. The test results are summarized in Table 4, and the table presents the average values of the test results, with the coefficients of variation for compressive strength and tensile strength being 0.08 and 0.13, respectively.

#### 2.2.2. Test Group

In this study, only two anchorage lengths, i.e., 3d and 5d (where d is the bar diameter), were adopted. These lengths were selected based on the following considerations. First, preliminary pull-out tests indicated that when the anchorage length exceeded 5d, the specimens tended to fail by bar fracture rather than bond slip, which prevents the evaluation of bond strength. Second, code provisions (e.g., GB 50010-2010 [21]) suggest that the development length for conventional deformed bars is generally above 5d; however, negative Poisson’s ratio bars/strands are expected to provide a confining effect that reduces the required anchorage length. Therefore, testing 3d (insufficient anchorage) and 5d (transitional length) effectively captures the bond performance envelope without prematurely inducing bar rupture. The test groups are detailed in Table 5.

#### 2.2.3. Specimen Fabrication

**Figure 7:**
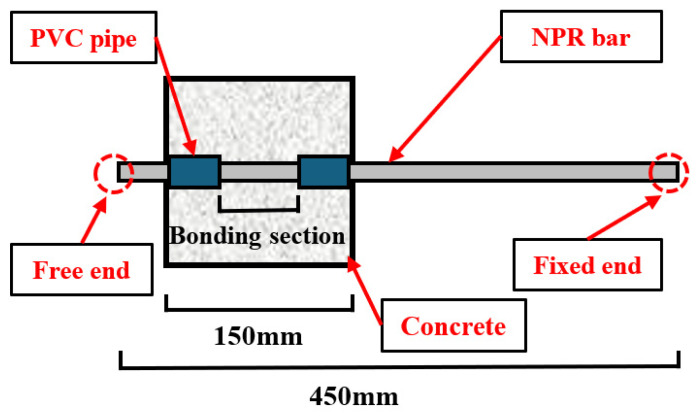
Adhesive slip mold production.

To ensure uniform anchorage, stress distribution and precise control over the anchorage length, the anchorage zone was centrally positioned within the concrete specimen. PVC pipes were used to sleeve the non-anchorage sections of the bar, thereby eliminating potential stress concentration effects at the specimen ends. The procedure involved placing the PVC sleeves over the intended non-anchored regions, securing them with foam adhesive, and sealing the tube ends to prevent cement paste infiltration during concrete casting and vibration. Standard 150 × 150 × 150 mm cubic molds were used for the specimens. To accurately fix the position of the bar, holes matching the outer diameter of the PVC pipe were drilled into the front and rear side walls of the molds. This study adopted two anchorage lengths: 3d and 5d. The construction of the specimen molds is detailed in Figure 7.

**Figure 8:**
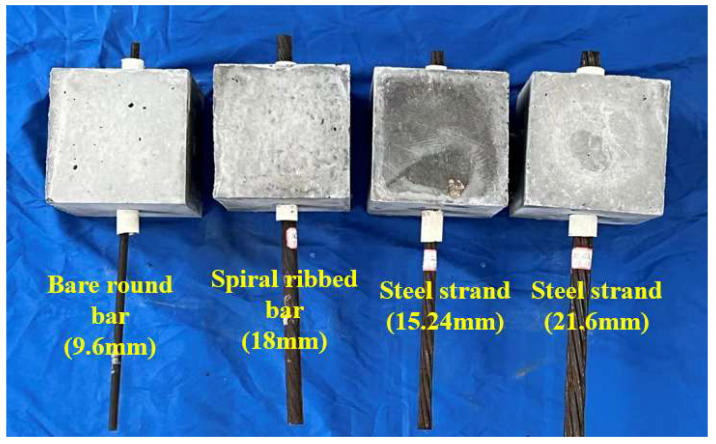
Pull-out test specimen.

The specimen preparation process involved the following steps: (1) All components were precisely weighed according to the designed mix proportions. (2) The steel bar was installed into the pre-drilled holes, ensuring stability and central positioning of the bonded segment. (3) During mixing, dry materials were combined and then mixed with water. (4) The mixture was poured into the molds, consolidated manually, vibrated on a vibrating table for approximately 30 s, and leveled. (5) The specimens were covered with a wet cloth, demolded after 24 h, and cured with water. The finished specimens are shown in Figure 8.

### 2.3. Test Method

**Figure 9:**
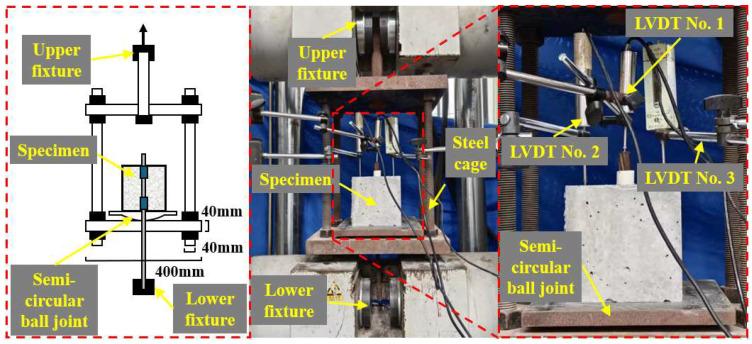
Center pull-out experimental setup.

A single-end center pull-out test was performed on the specimens using a universal testing machine. The setup incorporated a basket-type steel cage and a semi-spherical hinge plate placed above the lower bearing plate to ensure the bar remained perfectly aligned with the clamp center, preventing eccentric loading. The pull-out end of the specimen was clamped by the lower grip. The testing machine then drove the cage and the concrete block upward, causing the bar to be pulled out. A 30 mm range electronic displacement transducer (No. 1 LVDT) was installed at the free end to measure the bar’s slip. Two 50 mm range LVDTs (No. 2 and No. 3) were placed on the upper surface of the concrete cube to measure concrete displacement. The dimensions of the test setup are provided in Figure 9.

The test procedure was as follows: (1) Place the specimen on the bearing plate and clamp the bar’s free end. (2) Ensure full contact between the hinge plate and the specimen’s lower surface. (3) Mount the LVDTs for displacement monitoring. (4) Perform loading according to standard GB/T 50152-2012 [22]. The initial rate was 0.3 mm/min, which was increased to 1 mm/min upon reaching the ultimate load, given the NPR bar’s excellent elongation capacity.

To obtain the bond stress (*τ*)–slip (*s*) curve, the average bond stress (*τ*) was calculated as follows:

(1) $$\tau =\frac{F}{\pi dl_{a}}$$

where *F* is the applied load (N), *d* is the bar diameter (mm), and *l_a_* is the anchorage length (mm). The relative average slip (*s*) was calculated as follows:

(2) $$s_{u}=s_{u}-s_{r}$$

where *s_r_* is the bar displacement (from LVDT No. 1), and *s_c_* is the concrete displacement (the average of LVDTs No. 2 and No. 3).

## 3. NPR Pull-Out Test Results

The experimental results are listed in Table 6. Based on the bond–slip data characteristics, the slip stages for both the smooth NPR bars and the NPR steel strands are segmented into two distinct periods: Bond I and Bond II.

### 3.1. Failure Modes

The primary failure modes observed in the NPR bar/concrete pull-out tests were bar pull-out, concrete splitting, and splitting-accompanied pull-out. The bare round bar typically failed by pull-out; the spiral ribbed bar exhibited both pull-out and splitting failure modes; and the steel strand showed a mix of pull-out and splitting-accompanied pull-out failure.

#### 3.1.1. Pull-Out Failure

**Figure 10:**
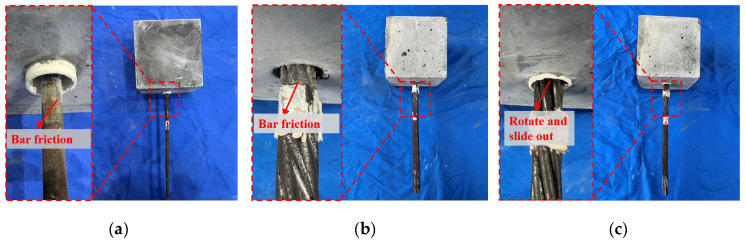
Pull-out destruction: (**a**) NC-R9.6D5T1; (**b**) NC-R18D3T2; (**c**) NC-R15.24D3T3.

The pull-out failure mode (see Figure 10) is typically observed in specimens characterized by smaller bar diameters, thicker concrete cover, or shorter anchorage lengths. Both bare round bar groups (NC-R9.6D3T1 and NC-R9.6D5T1) failed by pull-out failure. Figure 10a shows that the extracted bars had fine friction scratches and residual concrete powder. The spiral ribbed NPR bar group NC-R18D3T2 also failed by pull-out failure. Sufficient confinement from the concrete ensured that the bar only damaged the interfacial concrete ribs during extraction, leaving the concrete block intact without visible cracks. Shao et al. [11] reported a similar observation, noting that although no surface cracks appeared, the free end showed clear evidence of bar extraction.

Figure 10b shows that the extracted bars displayed concrete fragments and scratches. Pull-out failure was also recorded in the NPR steel strand group NC-R15.24D3T3. Similar to the spiral-ribbed bar’s pull-out failure, the concrete effectively confined the smaller diameter strand. As seen in Figure 10c, the extracted strand had only fine scratches and no significant concrete debris. Given the characteristics of the steel strand, failure involves the strand primarily rotating along the concrete’s helical teeth during extraction [23]. Furthermore, the extracted strands showed signs of unraveling (splaying), leading to observable strand expansion.

#### 3.1.2. Splitting Failure

**Figure 11:**
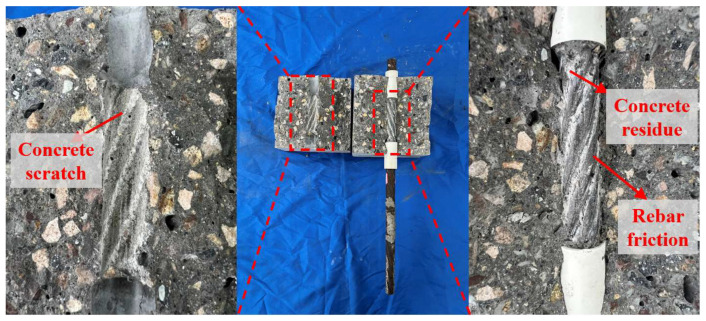
Split destruction (NC-R18D5T2).

The splitting failure mode (Figure 11) is frequently observed in specimens with larger bar diameters, smaller cover thickness, or longer anchorage lengths. In this test, a splitting failure occurred in the spiral ribbed NPR bar group NC-R18D5T2. For this group (NC-R18D5T2), the pull-out force was predominantly sustained by the shear strength between the spiral ribs and the concrete interface. Unlike the pull-out mode, the increased anchorage length (3d to 5d) led to a higher total load and mobilized greater shear stresses. This overburden overwhelmed the concrete’s lateral confinement capacity, initiating internal cracks that rapidly propagated along the bar surface and reached the specimen’s exterior, resulting in splitting failure and fracturing the specimen into pieces. Splitting failure is characterized by at least one through-thickness crack on the surface [11].

#### 3.1.3. Splitting–Pull-Out Failure

Splitting–pull-out failure is a transitional mode: splitting cracks develop as the pull-out force increases but do not immediately breach the surface until the moment of bar extraction, when sudden splitting occurs. This combined failure was primarily observed in the larger-bar diameter steel strand group, NC-R21.8D5T3.

**Figure 12:**
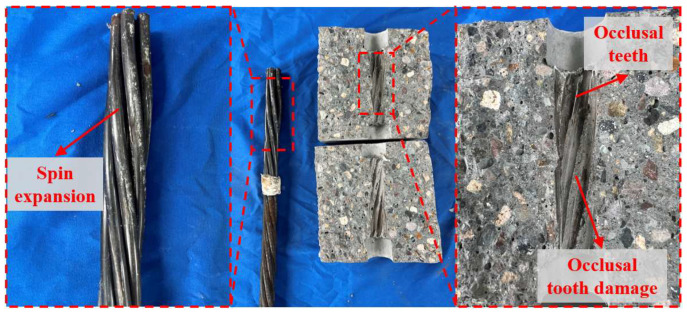
Splitting–pull-out destruction (NC-R21.8D5T3).

Figure 12 shows that the larger 21.8 mm strand diameter (NC-R21.8D5T3) resulted in a thinner concrete cover and a larger interface shear area compared to the 15.24 mm group, significantly increasing the shear stress. Additionally, the pull-out process generated a more substantial strand splaying and expansion effect. As the strand rotated and extracted, it continuously expanded, causing localized damage to the concrete teeth. Splitting failure occurred when the internal tensile stress exceeded the concrete’s strength. Post-failure inspection showed damaged concrete ribs and clear evidence of expansion resulting from the strand’s rotational movement.

**Figure 13:**
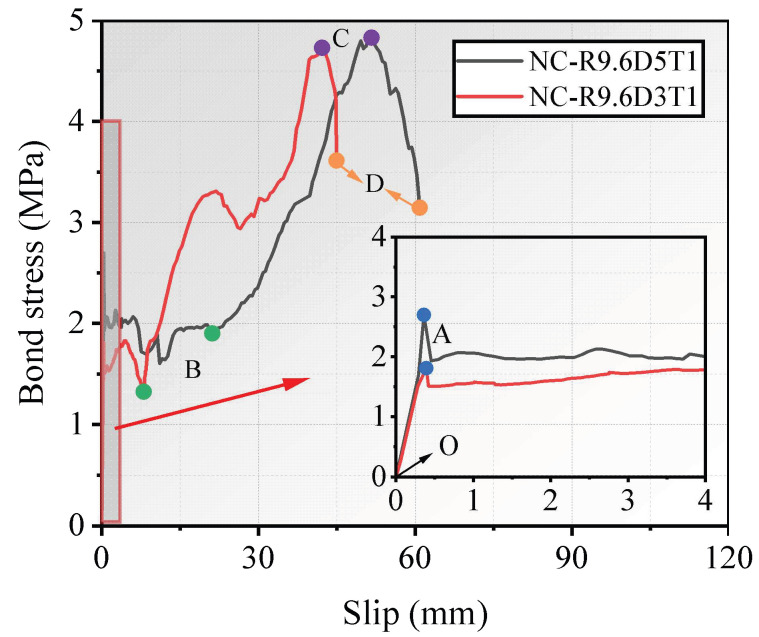
Bond–slip curves for bare round NPR bars.

Under tensile loading, the transverse contraction of conventional steel further promotes the opening of microcracks within the interfacial transition zone (ITZ), leading to premature degradation of bond stiffness and strength. For NPR bars, however, this micro-mechanism is fundamentally altered. During pull-out, the NPR material elongates longitudinally but expands transversely owing to its negative Poisson’s ratio. At the microscale, this expansion exerts active radial compressive stress on the surrounding ITZ, effectively densifying the porous zone, compressing inherent voids and microcracks, thereby increasing local contact stiffness. This mechanism explains the observed behavior: even the smooth NPR bar, despite its lack of ribs, exhibits a rising bond stress during the dilation-hardening stage (segment BC in Figure 13), as radial expansion progressively compacts the interface. The mechanical interlock of spiral-ribbed bars and strands benefits similarly, as the additional radial pressure delays shearing of concrete keys.

### 3.2. Bond–Slip Curves

**Figure 14:**
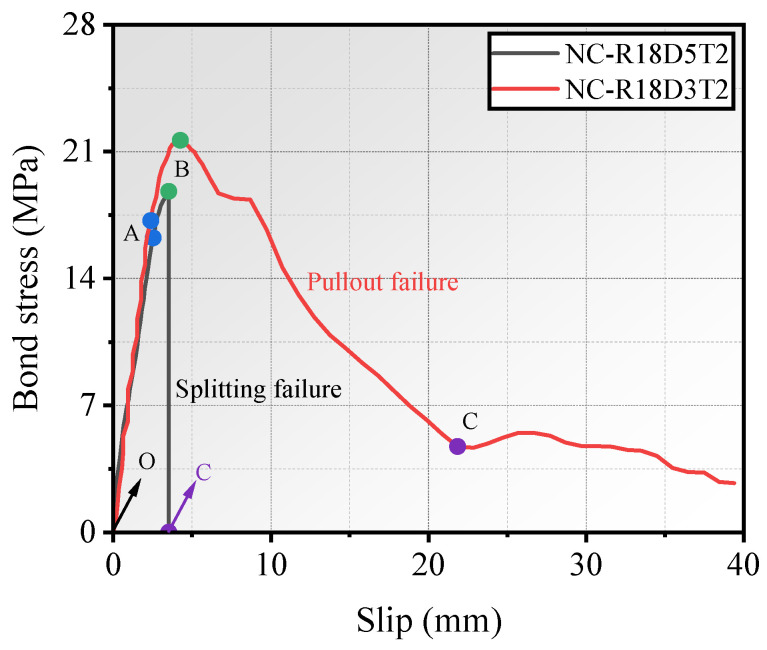
Bond–slip curves for spiral ribbed NPR bars.

**Figure 15:**
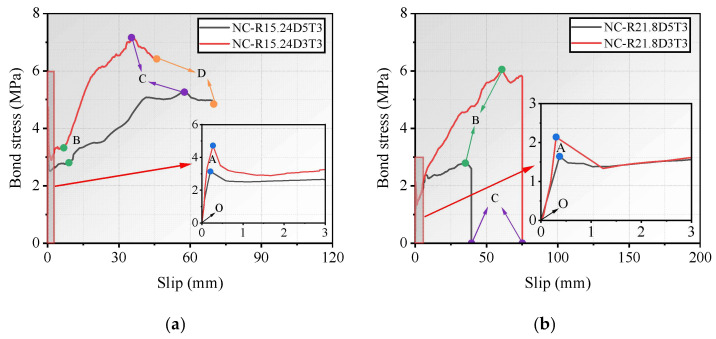
Bond–slip curves for stranded NPR bars: (**a**) NC-R15.24T3; (**b**) NC-R21.8T3.

The average bond–slip curves for smooth, spiral-ribbed, and stranded NPR bars are shown in Figure 13, Figure 14 and Figure 15. Two failure modes were observed—pull-out and splitting—depending on the bar type, anchorage length, and diameter. Rather than describing each bar separately, the overall pull-out response is synthesized here into a unified stage framework that highlights common initial behavior and subsequent mechanistic divergences.

In the micro-slip stage (OA), chemical adhesion holds the bond, the slip is minimal, and bond stress rises almost linearly for all bars. In the next stage (AB), the chemical bond breaks and load transfer shifts to friction or mechanical interlock, depending on the bar’s surface. Smooth NPR bars use only friction, so bond stress goes up and down with slip but does not drop significantly. Spiral-ribbed bars press into concrete for mechanical interlock, and bond stiffness slowly drops as the concrete between ribs shears off. Stranded tendons twist when pulled out [24]; 15.24 mm strands experience a slight rise in bond stress after the first drop, while thicker 21.8 mm strands have less twist and slip, so bond stress rises faster under mechanical interlock.

Beyond the slip stage, the curves diverge according to failure mode. When pull-out governs, smooth bars pass through a dilation-hardening stage (BC) where the bar’s slight dilation increases radial pressure and enhances confinement, followed by a descending stage (CD) where wear reduces interfacial friction and bond stress declines gradually. Spiral-ribbed bars with an anchorage length of 3d skip the separate dilation stage; after reaching a peak, bond stress declines as the concrete ribs are progressively crushed, often with slight recovery when the bar engages the next intact ribs. The 15.24 mm strands exhibit a strand-dilation stage (BC) in which untwisting of the wires increases the effective diameter and generates radial pressure, sustaining the bond through a combination of confinement, mechanical interlock, and friction, before frictional wear leads to a post-peak decline.

Splitting failure, observed in spiral-ribbed bars at a 5d anchorage length and in 21.8 mm strands, shortens the subsequent response. After a similar micro-slip and slip evolution, the increasing radial pressure from rib bearing or strand dilation surpasses the concrete tensile strength, and cracks propagate suddenly. In the larger strands, the bond stress drops slowly at first, and then abruptly to zero as the specimen splits.

This comparative synthesis eliminates the need to repeat the same initial stage descriptions for each bar type and directly links the divergent bond–slip mechanisms to surface geometry and confinement.

### 3.3. Influence of Different Factors on Bond Performance

#### 3.3.1. Reinforcing Bar Type

The bond–slip curves (Section 3.2), along with the data in Table 6 and Table 7, reveal distinct bond behaviors for the three NPR bar types. The T1 group (bare round bar) exhibited considerable ductility, with all specimens failing by pull-out. However, due to their smaller diameter (9.6 mm) and smooth surface, they demonstrated the lowest bond performance. Their average bond stresses in Stages I and II were 1.81–2.70 MPa and 4.73–4.83 MPa, respectively, which is the lowest among the three types. In contrast, the R18-T2 group (spiral ribbed bar) showed superior bond performance, with average bond stresses ranging from 18.81 to 21.61 MPa, attributed to the significant mechanical interlock provided by the ribs. Notably, the slip development for T2 bars was relatively limited and progressed slowly during pull-out. For the T3 group (steel strand), although the bar diameter and surface features are similar to T2 bars, the bond strength was markedly lower, at only 2.79–7.16 MPa. This is primarily because the helical wires of the tendon provide less effective bonding and anchorage compared to the solid ribs of T2 bars. During pull-out, the “strand-dilation” effect in T3 tendons accelerated the shearing of the concrete keys. Furthermore, pull-out typically occurred along the helical path of the wires, which further reduced the mechanical anchorage capacity.

Compared to conventional reinforcement, the NPR material significantly enhances deformation capacity. The bare round NPR bar achieved ultimate slips of 30–60 mm at peak bond stress, far exceeding the measured 10–15 mm reported for ordinary plain bars [25]. Similarly, the NPR steel strand attained ultimate slips of 30–70, compared to 3–20 mm for ordinary strands [24], indicating a substantial improvement in member ductility.

Compared with HRB400 bars, NPR bars exhibit a 15–20% lower bond strength under identical concrete strength and anchorage length. As shown in previous studies (Shao et al. [11]; Xiong et al. [12]; Zhou et al. [13]), the bond performance of NPR bars has been consistently evaluated through comparative quantification rather than through a direct model. Following this established approach, this study provides quantitative bond stress and slip models (see Section 4) and relative difference indices as the current state of knowledge.

#### 3.3.2. Bar Diameter

**Figure 16:**
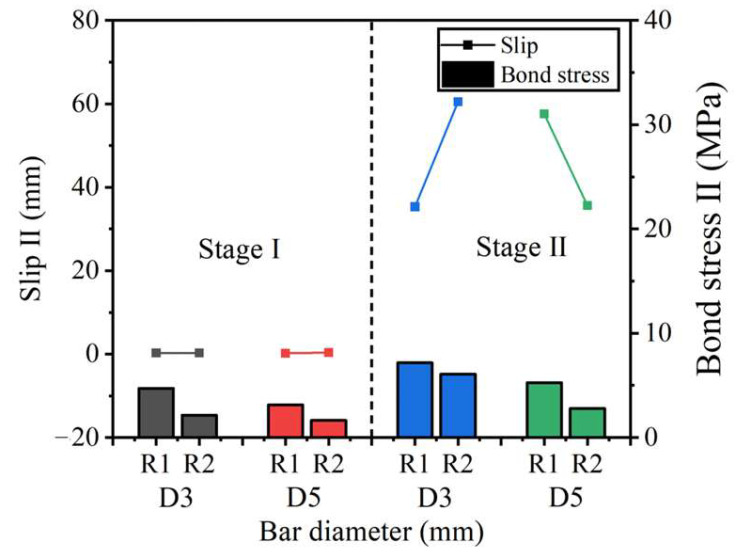
Influence of bar diameter on bonding properties.

The analysis of NPR strands with different bar diameters (Figure 16) shows that the diameter significantly affected their bond behavior with concrete. Increasing the diameter from R15.24 (15.24 mm) to R21.8 (21.8 mm) changed the failure mode from pull-out to splitting–pull-out. Consistent with established trends where larger bar diameters typically lead to reduced bond strength [26,27,28,29], the initial and residual bond stresses for the R21.8 strands were significantly lower than those for the R15.24 strands, with an average reduction of 15% to 55%. Furthermore, the larger diameter generally increased the slip at failure. For anchorage lengths of 3d and 5d, the slip increased by an average of 11.1%, 71.3%, and 85% across different measurement stages. An exception was observed in the Stage II slip for the 5d anchorage length group, where the slip decreased by 38.2% with the larger diameter.

The decrease in bond strength when the strand diameter increases from 15.24 mm to 21.8 mm can be decoupled into three interacting mechanisms: (1) Confinement softening: The larger diameter reduces the relative cover ratio, which, according to the thick-walled cylinder theory, lowers the radial confinement stiffness. This diminishes both frictional resistance and the bearing capacity of concrete keys. (2) Increases in stress non-uniformity: Larger bar diameters intensify bond stress concentration near the loaded end, causing a more non-uniform distribution and thus a lower average bond strength at failure. (3) Dilation-induced cracking: The greater splaying and expansion of the 21.8 mm strand generate higher splitting tensile stresses in the concrete teeth, directly triggering the shift from pull-out to splitting–pull-out failure.

#### 3.3.3. Anchorage Length

**Figure 17:**
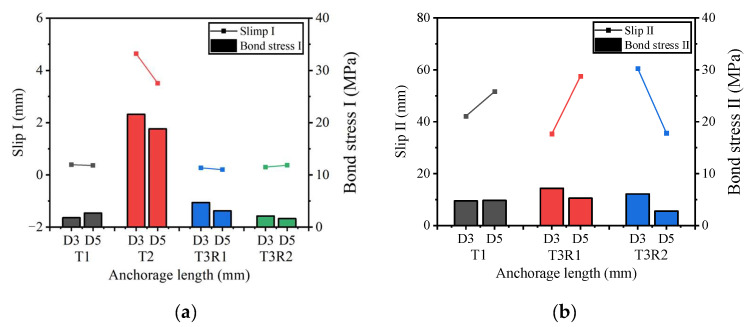
Effect of bond length on bond–slip performance: (**a**) Stage I; (**b**) Stage II.

A comparison of bond performance at different anchorage lengths (Figure 17) shows that, for all bar types except T1, the bond strength in Stages I and II decreased as the anchorage length increased from 3d to 5d. The average reductions were 12.98% for T2 bars and 34.96% for T3 bars. This aligns with the principle that longer anchorage lengths lead to a more non-uniform distribution of bond stress, resulting in a lower average strength at failure [11]. Conversely, for T1 bars, bond strength in Stages I and II increased by 49.17% and 2.11%, respectively, with longer anchorage. This is attributed to the friction-dominated bond mechanism in bare round bars; a longer bonded area provides greater cumulative frictional resistance. Regarding slip, increasing the anchorage length from 3d to 5d generally led to a decrease in Stage I slip (by 7.69% for T1, 24.36% for T2, and 25.93% for R15.24-T3) and an increase in Stage II slip (by 22.49% for T1 and 62.97% for R15.24-T3). The R21.8-T3 group was an exception, showing a 23.33% increase in Stage I slip and a 41.16% decrease in Stage II slip.

These failure modes lead to clear design limits. Splitting failure means spiral-ribbed bars need a minimum cover and a maximum anchorage length. Pull-out failure in smooth bars is ductile but calls for substantial embedment, which is not economical. Splitting–pull-out failure in large strands shows that radial expansion cannot be ignored. On the application side, the high slip capacity of NPR bars (30–70 mm) makes them well-suited for large-deformation situations like tunnel linings in squeezing rock or seismic joints. Also, for spiral-ribbed and stranded bars, an anchorage longer than 3d lowers the bond strength by 13–35%, so over-embedding should be avoided unless transverse reinforcement is added. These insights bridge lab bond–slip curves and practical design rules.

## 4. Prediction and Analysis of Key Parameters

### 4.1. Stage I Ultimate Strength and Slip

Empirical models were developed to predict the Stage I ultimate bond strength (*τ_u_*_1_) and corresponding slip (*s_u_*_1_) for plain NPR bars, considering concrete compressive strength (*f_cu_*), bar diameter (*d*), and anchorage length (*l_a_*):

(3) $$\tau_{u1}=0.21{\left(\frac{l_{a}}{d}+0.05f_{cu}\right)}^{1.26}$$

(4) $$S_{u1}=0.464{\left(\frac{l_{a}}{d}\right)}^{-0.157}$$

Given the limited number of spiral ribbed bar specimens in this study, a supplementary database was compiled from published pull-out tests [11,12,13] to enhance model reliability. The data selection criteria were: (a) only central pull-out tests; (b) only spiral ribbed bar NPR bars with varying rib spacings; and (c) only ordinary Portland cement concrete. The compiled database is presented in Table 1.

The model proposed by Li et al. [15] was adapted to fit the ultimate bond strength (*τ_u_*_2_) for spiral ribbed bars:

(5) $$\frac{\tau_{u2}}{f_{t}}=3.99\frac{d}{l_{a}}+1.3232$$

The fitted model is expressed as follows:

(6) $$\frac{\tau_{u2}}{f_{t}}=-0.4408\frac{d}{l_{a}}+0.5106\frac{f_{cu}}{f_{ts}}-0.4324$$

where the concrete tensile strength *f_t_* is calculated according to $f_{t}=0.3\;({f_{c}-8)}^{2/3}$ [28]. The coefficient of determination R^2^ was 0.6544. The corresponding slip at ultimate strength (*s_u_*_2_) was fitted considering the bar diameter, anchorage length, and concrete compressive and tensile strengths:

(7) $$s_{u2}=2.889-1.143d-0.54l_{a}+0.335f_{cu}-5.506f_{t}+0.0289\left(dl_{a}\right)$$

For NPR strands, models for Stage I ultimate strength (*τ_u_*_1_) and slip (*s_u_*_1_) were developed based on strand diameter, anchorage length, and concrete compressive strength:

(8) $$\tau_{u3}=3.74d^{-1.333}l_{a}^{-0.75}f_{cu}^{1.7}$$

(9) $$s_{u3}=0.1-0.078\frac{l_{a}}{d}+0.0042l_{a}+0.0036f_{cu}$$

### 4.2. Stage II Ultimate Strength and Slip

For plain NPR bars, the Stage II bond strength (*τ_u_*_1_) and slip (*s_u_*_1_) were fitted using the following models:

(10) $$\tau_{u1}=1.151{\left(\frac{l_{a}}{d}+0.5f_{cu}\right)}^{0.42}$$

(11) $$s_{u1}=3.14{\left(\frac{l_{a}}{d}+0.15f_{cu}\right)}^{1.1}$$

For NPR strands, the Stage II bond strength (*τ_u_*_3_) and slip (*s_u_*_3_) were fitted as follows:

(12) $$\tau_{u3}=10.51+1.104\frac{l_{a}}{d}-0.1207l_{a}$$

(13) $$s_{u3}=\frac{102-9.64d+1.13l_{a}}{1-0.14d+0.023l_{a}}$$

### 4.3. Model Performance

**Figure 18:**
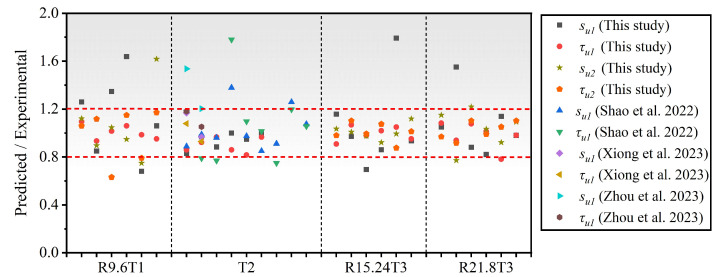
Model fitting effect [13,16,20].

The performance of the predictive models was evaluated by comparing predicted (Pre.) and experimental (Exp.) values, as shown in Figure 18. Overall, the models demonstrate good agreement with the data. Most data points from the present tests fall within a Pre./Exp. range of 0.8 to 1.2, with only a few outliers, primarily at higher predicted values. Predictions for the compiled database also showed satisfactory performance, with minor deviations likely attributable to variations in the source test conditions.

To quantify the prediction accuracy, the mean Pre./Exp. ratio and the Mean Absolute Percentage Error (MAPE) were calculated for Stage I/II bond stresses and slips, as summarized in Table 7 and Table 8. The models accurately predicted the behavior of T1 and T3 groups, with most Pre./Exp. ratios close to 1.00 and MAPE values generally below 5%. The slightly higher MAPE for strands may be related to uncertainties inherent in their material properties, manufacturing, and specimen preparation, which could affect confinement during pull-out.

The prediction accuracy for the T2 group was lower, with MAPE values exceeding 6% for both bond stress and slip. This indicates that the bond–slip behavior of spiral ribbed bars is influenced by a more complex set of factors under varying conditions, necessitating further investigation and model refinement.

**Figure 19:**
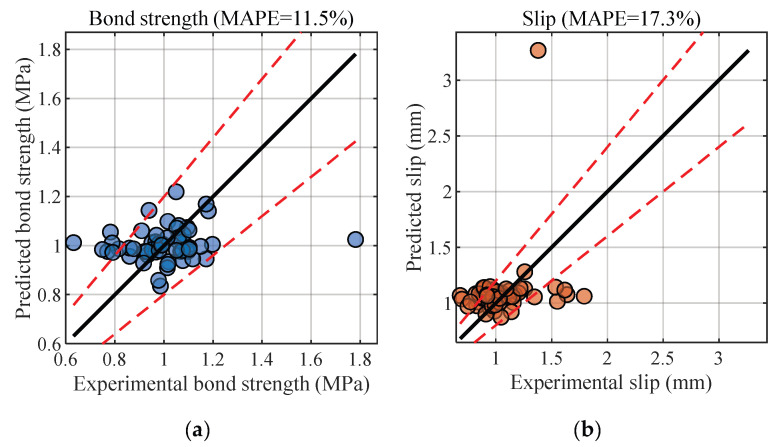
Cross-validated predicted results: (**a**) bond strength; (**b**) slip.

To independently evaluate the predictive capability of the proposed models, a five-fold cross-validation was performed on the combined dataset. Figure 19a compares the cross-validated predicted bond strengths against experimental values, and Figure 19b compares the same for slip. For bond strength, the MAPE is 11.5%, the mean Pre./Exp. ratio is 1.022 (σ = 0.162), and 83.6% of points fall within the 0.8–1.2 band. For slip, the MAPE is 17.3%, the mean Pre./Exp. ratio is 1.064 (σ = 0.267), and 67.3% fall within 0.8–1.2. These cross-validation results provide a more rigorous assessment of model generalizability than a simple comparison with data compiled from the literature, satisfying the requirement for independent dataset validation.

## 5. Conclusions

This study investigated the bond behavior of NPR material and concrete, including the bare round NPR bar, NPR steel strand, and spiral ribbed NPR bars. The main conclusions are as follows:

(1) The stress–strain curves of NPR bars primarily consist of an elastic stage followed by a hardening stage. The elongation at break reached 51.2%, 20.8%, and 20% for the bare round bar, steel strand, and spiral ribbed bar, respectively, and it is much taller than regular steel bars. The elastic modulus of these materials is approximately 150 GPa.
(2) In the bond–slip tests, bare round bar, spiral ribbed bar with a 3d anchorage length, and 15.24 mm diameter steel strands all failed by pull-out. Post-failure inspection revealed concrete powder and scratching on the bar surfaces, with strands also exhibiting slight dilation. In contrast, spiral ribbed bars with a 5d anchorage length failed by concrete splitting, characterized by sudden concrete cracking and abundant debris on the bar surface. Steel strands with a 21.8 mm diameter exhibited a splitting–pull-out failure mode accompanied by pronounced strand dilation.
(3) Bond–slip curves indicated distinct behaviors: The bare round bar exhibited large slips (42.15–51.63 mm) and a smooth curve, with a corresponding bond strength of 4.73–4.83 MPa. Spiral ribbed bars achieved high bond strength (18.81–21.61 MPa) within a small slip range (3.51–4.64 mm), showing a near-linear increase. Stranded NPR bars showed moderate bond strength (2.79–7.16 MPa) and large slips (35.32–60.50 mm), with a gently rising curve.
(4) The effect of anchorage length on bond strength depended on the bar type. The bond strength of spiral ribbed bars and steel strands decreased by an average of 12.98% and 34.96%, respectively, as the anchorage length increased from 3d (three times the diameter of the rebar) to 5d. Conversely, the bond strength of the bare round bar increased with longer anchorage. Furthermore, increasing the strand diameter from 15.24 mm to 21.8 mm significantly reduced bond performance (by 15% to 55%) and changed the failure mode from pull-out to splitting–pull-out.
(5) The proposed empirical models accurately predict the bond–slip behavior for bare round bars and steel strands, with MAPE < 5%, but show lower accuracy for spiral ribbed bars, with MAPE > 6%. A five-fold cross-validation performed on the combined dataset (55 points) provides an independent assessment of model generalizability, yielding an overall MAPE of 11.5% for bond strength and 17.3% for slip, with 83.6% and 67.3% of predicted-to-experimental ratios falling within the 0.8–1.2 range, respectively. Cross-validation satisfies the requirement for independent dataset validation and highlights that further refinement is needed for slip prediction and spiral ribbed bars.

## 6. Limitations and Future Work

It should be noted that the present experimental program was limited to two anchorage lengths (3d and 5d). While these parameters are sufficient to demonstrate the bond performance of negative Poisson’s ratio bars/strands compared with conventional steel, the results may not be directly extrapolated to very short (≤2d) or very long (≥10d) anchorage zones, nor to larger diameters (≥20 mm). Further experimental studies covering a broader range of diameters and anchorage lengths are necessary to establish a full design model.

## Figures and Tables

**Table 1:** NPR pull-out experimental research data.

Specimen	Concrete Type	*f_cu_* (MPa)	Bar Diameter (mm)	NPR Type	Bond Stress (MPa)	Bond Slip (mm)	Failure Mode	Source
NPR 2.5% 3d	UHPC	153.2	16	T4	55.12	1.53	Pull-out	Lu et al. [10]
NPR 2% 3d	UHPC	139.6	16	T4	46.29	1.29	Pull-out	
NPR 1% 3d	UHPC	128.5	16	T4	43.08	0.61	Splitting–Pull-out	
NPR 0–3d	UHPC	86.2	16	T4	31.34	0.38	Splitting	
NPR 2.5% 4d	UHPC	153.2	16	T4	47.16	1.99	Pull-out	
NPR 2.5% 5d	UHPC	153.2	16	T4	40	1.64	Pull-out	
NPR 2.5% 6d	UHPC	153.2	16	T4	37.1	1.42	Pull-out	
NPR 2.5% 3d	UHPC	153.2	16	T4	47.43	0.85	Pull-out	
NPR 2.5% 3d	UHPC	153.2	16	T4	50.51	0.96	Pull-out	
NPR 2.5% 3d	UHPC	153.2	16	T4	51.46	1.06	Pull-out	
NPRA 2.5% 3d	UHPC	153.2	16	T4	50.76	0.88	Pull-out	
NPRB 2.5% 3d	UHPC	153.2	16	T4	55.11	2.51	Pull-out	
A	UHPC	133.97	16	T4	52.59	0.5	Pull-out	Lu et al. [3]
RR	UHPC	133.97	16	T4	50.56	0.62	Pull-out	
B	UHPC	133.97	16	T4	49.42	0.8	Pull-out	
C	UHPC	133.97	16	T4	44.62	1.16	Pull-out	
D	UHPC	133.97	16	T4	43.1	1.51	Pull-out	
2	UHPC	133.97	16	T4	37.18	1.77	Pull-out	
E	UHPC	133.97	16	T4	45.03	0.61	Pull-out	
F	UHPC	133.97	16	T4	51.74	0.95	Pull-out	
G	UHPC	133.97	16	T4	53.99	1.11	Pull-out	
H	UHPC	113.34	16	T4	40.78	0.23	Splitting–Pull-out	
I	UHPC	121	16	T4	41.6	0.58	Splitting–Pull-out	
J	UHPC	125.4	16	T4	45.68	0.7	Splitting–Pull-out	
K	UHPC	133.97	16	T4	35.01	0.42	Splitting	
L	UHPC	133.97	16	T4	43.1	0.7	Splitting–Pull-out	
M	UHPC	133.97	16	T4	45.06	1.52	Pull-out	
N	UHPC	133.97	16	T4	54.25	0.44	Pull-out	
O	UHPC	133.97	12.6	T4	41.33	2.76	Pull-out	
P	UHPC	133.97	12	T4	50.06	2.11	Pull-out	
Q	UHPC	133.97	12	T4	55.42	1.45	Pull-out	
N08/5 d/30	NC	34.2	8	T2	8.95	4.84	Pull-out	Shao et al. [11]
N18/5 d/30	NC	34.2	18	T2	13.29	3.56	Splitting	
N18/7 d/30	NC	34.2	18	T2	10.59	2.96	Splitting	
N08/5 d/40	NC	49.2	8	T2	8.81	3.04	Pull-out	
N18-5 d-40	NC	49.2	18	T2	14.30	3.50	Splitting	
N18/7 d/40	NC	49.2	18	T2	12.34	3.22	Splitting	
N08/5 d/50	NC	63.4	8	T2	27.86	5.10	Pull-out	
N18/5 d/50	NC	63.4	18	T2	17.43	3.07	Splitting	
N18/7 d/50	NC	63.4	18	T2	16.12	2.97	Splitting	
C3-17-5d	NC	43.3	17	T2	12.6	1.83	Pull-out	Xiong et al. [12]
C6-17-5d	NC	43.3	17	T2	14.63	2.21	Splitting	
N-5d-0%	NC	36.7	18	T2	9.59	2.24	Splitting	Zhou et al. [13]
N-7d-0%	NC	36.7	18	T2	8.41	2.3	Splitting	

T2 represents the spiral-ribbed NPR bar; T4 represents the ribbed NPR bar.

**Table 2:** Test results of NPR bars.

Types of Reinforcing Steel	Bar Diameter (mm)	Yield Strength (MPa)	Ultimate Strength (MPa)	Elongation (%)	Modulus of Elasticity (GPa)
Bare round bar	9.6	362	806	51.2	150
Spiral ribbed bar	18	808	897	20.8	147
Steel strand	15.24	784	854	19.9	154
	21.6	773	844	20.1	141

**Table 3:** Concrete mixing ratio.

Mix.	Raw Material (kg/m^3^)						
	Cement	FA	GBFS	Sand	Stone	Water	Water Reducer
C50	428	44	83	653	1022	150	6.7

**Table 4:** Basic performance of concrete.

Mix.	*f_cu_* (MPa)		*f_ts_* (MPa)		Slump (mm)
	7-Days	28-Days	7-Days	28-Days	
C50	37.25	51.42	1.37	2.89	151

**Table 5:** Test specimen group.

Specimen ID	Bar Diameter (mm)	Anchorage Length (mm)	NPR Type	Number of Components
NC-R9.6D3T1	9.6	28.8	Bare round bar	3
NC-R9.6D5T1	9.6	48	Bare round bar	3
NC-R18D3T2	18	54	Spiral ribbed bar	3
NC-R18D5T2	18	90	Spiral ribbed bar	3
NC-R15.24D3T3	15.24	45.72	Steel strand	3
NC-R15.24D5T3	15.24	76.2	Steel strand	3
NC-R21.8D3T3	21.8	65.4	Steel strand	3
NC-R21.8D5T3	21.8	109	Steel strand	3

**Table 6:** Test results of bond stress and failure mode.

Specimen ID	Bond Stress I (MPa)				Bond Stress II (MPa)				Failure Mode		
	I	II	III	Ave.	I	II	III	Ave.	I	II	III
NC-R9.6D3T1	1.67	1.96	1.8	1.81	4.45	4.54	5.21	4.73	P	P	P
NC-R9.6D5T1	2.54	2.73	2.83	2.70	4.22	6.13	4.14	4.83	P	P	P
NC-R18D3T2	22.97	21.43	20.44	21.61	-	-	-	-	P	P	P
NC-R18D5T2	19.17	20.19	17.06	18.81	-	-	-	-	S	S	S
NC-R15.24D3T3	5.09	4.33	4.73	4.72	7.47	6.64	7.37	7.16	P	P	P
NC-R15.24D5T3	3.1	3.01	3.32	3.14	4.8	5.89	5.09	5.26	P	P	P
NC-R21.8D3T3	2.03	2.34	2.04	2.14	6.21	6.56	5.46	6.08	SP	SP	SP
NC-R21.8D5T3	1.48	1.92	1.53	1.64	2.95	2.78	2.65	2.79	SP	SP	SP

Note: P represents pull-out failure; S represents splitting failure; SP represents splitting–pull-out failure; T1 represents plain NPR bar; T2 represents spiral-ribbed NPR bar; T3 represents NPR steel strand.

**Table 7:** Test results of bond slip.

Specimen ID	Bond Slip I (mm)				Bond Slip II (mm)			
	I	II	III	Ave.	I	II	III	Ave.
NC-R9.6D3T1	0.31	0.46	0.29	0.39	38.06	47.61	40.77	42.15
NC-R9.6D5T1	0.22	0.53	0.34	0.36	54.32	68.74	31.82	51.63
NC-R18D3T2	4.98	4.26	4.67	4.64	-	-	-	-
NC-R18D5T2	3.45	3.64	3.44	3.51	-	-	-	-
NC-R15.24D3T3	0.21	0.25	0.35	0.27	34.32	35.22	36.43	35.32
NC-R15.24D5T3	0.25	0.12	0.23	0.20	62.8	58.21	51.68	57.56
NC-R21.8D3T3	0.31	0.21	0.37	0.30	52.86	78.78	49.85	60.50
NC-R21.8D5T3	0.43	0.31	0.36	0.37	34.85	39.06	32.93	35.6

**Table 8:** Model fit data.

Group	Specimen ID	Bond Slip I		Bond Stress I		Bond Slip II		Bond Stress II	
		Pre. /Exp.	MAPE (%)	Pre. /Exp.	MAPE (%)	Pre. /Exp.	MAPE (%)	Pre. /Exp.	MAPE (%)
T1	NC-R9.6D3T1	1.00	0.12	1.01	0.69	1.01	0.73	1.00	0.37
	NC-R9.6D5T1	1.00		1.00		1.00		1.00	
T2	NC-R18D3T2	0.89	6.39	0.91	10.49	N/A			
	NC-R18D5T2	0.98		0.88					
	Shao et al. [8]	1.03	12.45	1.07	22.24				
	Xiong et al. [13]	1.07	10.03	1.00	7.49				
	Zhou et al. [14]	1.37	37.00	1.12	11.64				
T3	NC-R15.24D3T3	0.90	3.45	0.98	3.67	1.01	0.65	1.02	2.49
	NC-R15.24D5T3	1.08		1.01		1.00		0.98	
	NC-R21.8D3T3	1.09		1.03		1.00		0.99	
	NC-R21.8D5T3	0.95		0.92		1.01		1.05	

## Data Availability

The original contributions presented in this study are included in the article. Further inquiries can be directed to the corresponding authors.
